# Ciprofloxacin pre-exposure influences individual cell MIC and heteroresistance of bacteria inside microfluidic droplets

**DOI:** 10.1038/s41598-025-17157-0

**Published:** 2025-09-25

**Authors:** Shakeel Ahmad, Ilona P. Foik, Paweł Jankowski, Adam Samborski, Shreyas K. Vasantham, Piotr Garstecki

**Affiliations:** Institute of Physical Chemistry, Polish Academy of Sciences, Warsaw, Poland

**Keywords:** Antibiotics, Antimicrobial resistance

## Abstract

Bacterial cells in general present different phenotypes even within the monoclonal populations. Some phenotypes may offer selective advantages under antibiotic stress. In clinical practice such advantage may lead to selection of resistant phenotypes and to treatment failure. Improving treatment regimens thus requires thorough understanding of the underlying cause of developing phenotypic variations within bacterial populations exposed to antibiotics. Here, we use droplet microfluidics to study the response of individual bacterial cells. We investigate how a short-term exposure of ciprofloxacin or streptomycin at the sub-minimum inhibitory concentration levels changes the single-cell distribution of susceptibility on subsequent exposure to either of the antibiotics. We used three different concentrations of pre-exposure for each antibiotic on *Escherichia coli* MG1655 and followed with re-exposure of the same bacterial cells individually in microfluidic droplets. Experiments with various concentrations of re-exposure on single cells showed that pre-exposures to sub-lethal levels of ciprofloxacin can elevate the individual cell MIC and the level of heteroresistance to ciprofloxacin. Exposure to quarter and half MIC of ciprofloxacin in separate experiments increased the heteroresistance of pre-exposed cells by more than 20-fold on subsequent treatment with ciprofloxacin. Interestingly, the same ciprofloxacin pre-exposure pattern made the cells more fragile and, in some cases, decreased the individual MIC values to streptomycin. The results in our study can be very important in developing new better treatment regimen, addressing the wider problem of antibiotic resistance.

**Supplementary Information:**

The online version contains supplementary material available at 10.1038/s41598-025-17157-0.

## Introduction

Antibiotics have been humanity’s primary defense against bacterial infections for decades, ever since their introduction in the early 1900s. However, reports of resistance began to appear as early as the 1930s, and today, antibiotic resistance has become a global health threat requiring urgent attention^1^. Simply speaking, resistance occurs when bacteria evolve survival mechanisms that render an effective specific drug concentration ineffective, making infections harder or even impossible to treat^2^. This can lead to increased medical costs, faster spread of diseases, severe illnesses, and higher mortality rates^3,4^. The situation is particularly alarming because the peak “golden age” of antibiotic discovery ended in the mid-1950s, and since then, the discovery and development of new antibiotics has gradually declined^5^. While resistant cells may naturally exist within a bacterial population or develop over time, improper and inefficient antibiotic use significantly accelerates the process^6^. According to World Health Organization estimates, drug-resistant diseases could cause 10 million deaths annually by 2050 and can surpass all other causes of mortality in the world, if substantial action is not taken^7,8^. Therefore, a comprehensive investigation is essential to understand the mechanisms of resistance, develop new antibiotics, and ensure the judicious use of existing ones.

Antibiotics work through various mechanisms, such as inhibiting DNA replication (e.g., ciprofloxacin) or protein synthesis (e.g., streptomycin). Multiple studies have shown that the bactericidal antibiotic activity is often accompanied by the generation of highly unstable reactive oxygen species (ROS) which cause oxidative damage to cellular DNA, proteins and membrane lipids^9^. However, most bacteria can tolerate very low levels of damage from radical species due to the evolution of various defense mechanisms^9,10^, and sometimes low-level ROS can be even beneficial to the bacteria, imparting certain protective functions^11^. When the exposure time or concentration of an antibiotic is insufficient to kill the bacterial cells, and some cells manage to evade its effects, there is a considerable chance that the surviving cells will show altered responses to future antibiotic treatments. The kind of response by bacteria on low level antibiotic exposure can be immense with reports showing induction of genes coding for virulence factors^12^, increased rates of conjugation and mutation via SOS response^13^, gene upregulation and DNA transfer occurrences^14^ etc. In general, the overall impact of low-level antibiotic exposure can be classified into at least three distinct categories: (I) resistance selection- allowing pre-existing resistant bacteria to proliferate or selecting for de novo resistance, (II) generating genetic and phenotypic heterogeneity- altering the rate of adaptive evolution and promoting development of resistance, and (III) signaling molecules- acting as mediators of physiological activities, such as virulence and gene expression^15^. Cumulatively, the low-level selective pressure exerted by the sub-lethal antibiotic concentrations is believed to play a crucial role in altering antibiotic susceptibility patterns, influencing bacterial selection, evolution, and treatment outcomes^10,16^.

To assess the interaction between an antibiotic and its target bacteria, an antibiotic susceptibility testing (AST) is performed. A key parameter in AST is the minimum inhibitory concentration (MIC), defined as the lowest concentration of an antibiotic that completely inhibits the visible growth of a specific bacterial strain^17^. The gold-standard methods for measuring MIC include disc diffusion, broth dilution, and the E-test, but these techniques tend to be time-consuming, require large sample volumes, are reagent-intensive and laborious^18^. Moreover, these methods rely on population-average data, failing to capture the behavior of individual cells, which is critical for understanding heteroresistance within a bacterial population at the smallest scale^19,20^.

Broadly defining, heteroresistance is a phenomenon where seemingly isogenic sub-populations of bacteria exhibit a wide range of susceptibilities to a given antibiotic^21^. Variability in bacterial responses to antibiotics can arise from a combination of stochastic processes, epigenetic factors, and genetic diversity^22–24^. Heteroresistance is known to be implicated in chronic infections recurrent infections, and in the infections with increased mortality rates^21^. While most cases of heteroresistance in clinical isolates are attributed to genetic heterogeneity, there is growing interest in studying phenotypic heterogeneity and its role in antibiotic treatment failure^25^. Studies suggest that the sensitive and precise detection of phenotypic heteroresistance is crucial for designing successful treatment regimens as variations in the pattern and the ratio of heterogenous phenotypes may affect the biological functions of the entire population^26^. Advanced single-cell approaches, such as microfluidics, have already proven to be excellent tools for studying heteroresistance by helping to capture the behavior of individual cells^27,28^.

A subtype of microfluidics, droplet microfluidics, have been widely used in research and industry to monitor the growth of individual bacterium within nanoliter droplets under variety of experimental conditions, including antibiotic exposure^28,29^. This technique allows for the encapsulation of single cells inside individual droplets through stochastic confinement, offering numerous advantages such as controlled cell densities, portability, ease of usage, cost-effectiveness, reproducibility, high throughput, and efficiency with reagents and samples^30–32^.

In our work, we used droplet microfluidics to investigate how the pre-exposure to ciprofloxacin (CIP) or streptomycin (STR) at sub-minimum inhibitory concentrations or in short sub-MIC (0.125X MIC, 0.25X MIC, 0.5X MIC) levels influences the response of individual bacterial cells to subsequent exposures of antibiotics in terms individual cell MICs. Pre-exposure concentrations of antibiotics were chosen, and the cells were exposed during their early growth phase. Following this, thousands of nanoliter water-in-oil microfluidic droplets were generated, each encapsulating a just one bacterium along with an appropriate antibiotic concentration. Unlike population-level studies, this approach allowed us to culture individual bacteria independently, eliminating cell-to-cell crosstalk and enabled precise monitoring of growth in the presence of antibiotics, thus helping to elucidate the heteroresistance pattern^31,33^.

Data analysis revealed that pre-exposure to ciprofloxacin at 0.25X MIC and 0.5X MIC resulted in more than a 20-fold increase in heteroresistance during AST with ciprofloxacin. Additionally, the minimum antibiotic concentration required to completely inhibit the bacterial growth in droplets was also elevated. For streptomycin susceptibility, the pre-exposure to 0.5X MIC of ciprofloxacin resulted in a 15-fold increase of heteroresistance. Further molecular and genetic studies within droplets could provide insights into the mechanisms underlying this differential behavior, suggesting that the heterogeneity can be regulated and functional rather than merely a reflection of noisy biochemistry^34^.

## Analysis terms and parameters

Previously multiple single-cell-based AST studies have already been performed using different approaches and gave rise to multiple definitions of MIC concerning single cells. Artemova et al. defined single-cell MIC (scMIC) as the measured MIC values at very low initial cell densities^35^. In another publication by Pacocha et al., scMIC was referred to the average value of individual cell MICs measured, when the cells are singly encapsulated in droplets^36^. Lyu et al. monitored the growth inhibition of single cells inside microfluidic droplets^37^ while Baltekin et al. imaged the multiplication of single cells inside microfluidic traps^38^ in the presence of antibiotic. To avoid confusion, we introduced new a term individual MIC (*iMIC*) in our previous publication^39 ^based on the observation of phenotypic growth inhibition of individual bacterium inside microfluidic droplets. The *iMIC* is defined as minimum antibiotic concentration required to completely inhibit the visible growth of an individual bacterium encapsulated within the microfluidic droplet. Conceptually, the *iMIC* is different from how Artemova et al. or Pacocha et al. defined, however, it is similar what Lyu et al. and Baltekin et al. proposed except incubation time used in the studies.

While dealing with studies involving droplets for single-cell AST, it is crucial that only one bacterium gets encapsulated per droplet. To ensure this, during generation of droplets of required volume, the concentration of bacteria is pre-adjusted according to the Poisson distribution^28,36,40^. For the droplets having 1 nl volume, a bacterial concentration of 1 × 10^5^ CFU/mL resulted in approximately 90% empty droplets, 9% with one bacterium per droplet, and the rest with two or more bacteria.

**Fig. 1:**
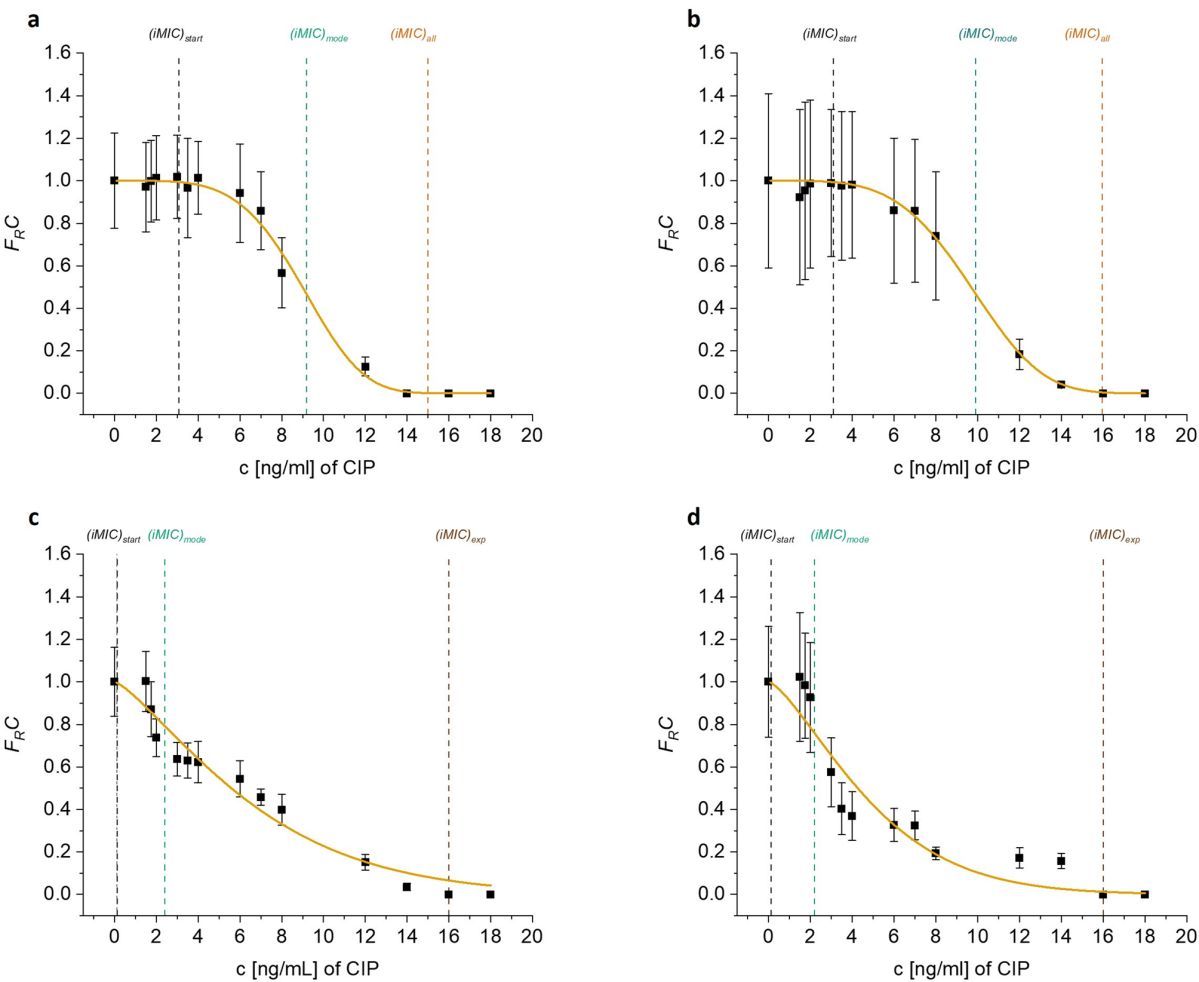
Plot depicts the resistance profiles and the Gompertz fitting curves for positive fraction of droplets, *F*_*R*_*C*, as a function of antibiotic concentration, c, from lowest to highest. The susceptibility tests were performed independently against ciprofloxacin (CIP) on (**a**) control (unexposed) sample and (**b**) bacterial sample pre-exposed with 0.125X MIC of ciprofloxacin, (**c**) 0.25X MIC of ciprofloxacin (**d**) 0.5X MIC of ciprofloxacin. Each black solid square data points are average of three independent biological replicates and error bars represent the standard deviations. It was not possible to determine *(iMIC)*_*all*_ for (**c**) and (**d**) from Gompertz fitting, hence the experimentally determined concentration for inhibition of all cells, *(iMIC)*_*exp*_, is represented instead. More information about this is in the manuscript text and Table 1. The resistance profile figures for other pre-exposures can be found in the supplementary file.

We denote the total number of droplets by *N(c)*, and the number of droplets showing detectable bacterial growth (positive droplets) by *N*_*+*_*(c)*^39^. After normalizing the number of positive droplets with respect to the sample without any antibiotics, a Gompertz fitting was performed for the positive droplet fractions, *F*_*R*_*(c)*, as a function of antibiotic concentration from lowest to highest^28,41,42^. The above fitting was done by using the average values of *N(c)* and *N+(c)* obtained from three independent biological replicates (Figure 1). A t-test was also performed on the Gompertz fitting of each pre-exposed sample with respect to the control (unexposed) sample. Furthermore, the probability density distribution, *p(iMIC)*, representing the likelihood of droplets showing specific *iMIC* values is also calculated as the negative first derivative of *F*_*R*_*(c)*. This distribution allows us to visualize the spread of *iMIC* values across the range of antibiotic concentrations.

The droplets generated from the sample without any antibiotic will theoretically show growth in all successful encapsulations having the maximum possible number of positive droplets, *N*_*+*_*(0)*, and the number of positive droplets in subsequent samples is normalized with respect to *N*_*+*_*(0)*. Furthermore, the lowest antibiotic concentration that inhibits bacterial growth in > 95% droplets as compared to *N*_*+*_*(0)*, is denoted by *(iMIC)*_*total*_, while the concentration needed to completely inhibit the growth of bacteria in all droplets of a sample is denoted by *(iMIC)*_*all*_. *(iMIC)*_*exp*_ is analogous to traditional MIC and is experimentally determined value, referred to as the minimum antibiotic concentration that completely inhibited the visible growth of bacteria in all screened droplets, as observed under microscope. Another insightful parameter is (*iMIC)*_*start*_ which is the minimum concentration where the first drop in growth is observed, as indicated by the Gompertz fitting curve. *(iMIC)*_*mode*_ is the most frequently observed *iMIC* value, denoted by the maximal point on the *p(iMIC)* distribution curve, refers to the inflection point on the Gompertz fitting. *(iMIC)*_*mode*_ represents the most probable antibiotic concentration at which major number of individually cultured bacteria exhibit their *iMIC* value, when allowed to grow in droplets. These parameters provide a very comprehensive framework to characterize antibiotic susceptibility at single-cell level, enabling precise assessment of heterogeneity within bacterial populations.

## Results

### Minimum inhibitory concentration (MIC)

Using broth dilution method, the MICs of ciprofloxacin and streptomycin for *Escherichia coli* MG1655 were found to be 12 ng/ml and 3 µg/ml, respectively (Supplementary table ST1; Supplementary figure SF1). The MICs of all pre-exposed samples also remained the same as with the control (unexposed) sample for both ciprofloxacin and streptomycin.

### Effect of pre-exposure on (iMIC)_mode_, (iMIC)start, (iMIC)_all_, (iMIC)_exp_, and p(iMIC)

To understand how the pre-exposure with an antibiotic affects the *Escherichia coli* MG1655 sensitivity on subsequent exposure we calculated *(iMIC)*_*mode*_, *(iMIC)*_*start*_, *(iMIC)*_*all*_.

As previously mentioned, (*iMIC)*_*mode*_ refers to the most probable antibiotic concentration at which the highest number of positively encapsulated droplets showed growth inhibition. *(iMIC)*_*mode*_ during AST of control (unexposed) sample against ciprofloxacin was found to be 9.2 ng/ml, while it was 9.5 ng/ml, 10 ng/ml and 9.5 ng/ml for the 0.125X, 0.25X and 0.5X MIC of streptomycin pre-exposures respectively. However, a 4X decrease in *(iMIC)*_*mode*_ value, as compared to unexposed cells, was observed in the samples pre-exposed with 0.25X MIC and 0.5X MIC of ciprofloxacin, implying that the majority of cells were either already in dying stage due to the pre-exposure at the instant of encapsulation or they were in a state where even very low concentration (around the *(iMIC)*_*mode*_ values i.e. 2.4 ng/ml and 2.2 ng/ml) of ciprofloxacin was enough to cause inhibition (Table 1).

Moreover, *(iMIC)*_*mode*_ during the AST of control (unexposed) sample against streptomycin was calculated to be 1.1 µg/ml and in comparison, the *(iMIC)*_*mode*_ was increased to 1.5 µg/ml in sample exposed with 0.125X MIC of ciprofloxacin. Pre-exposures with all the other concentrations of either ciprofloxacin or streptomycin decreased the *(iMIC)*_*mode*_ in comparison to respective control (unexposed) sample, the least one being 0.33 µg/ml for 0.5X MIC of ciprofloxacin pre-exposure (Table 2).

The most notable change in (*iMIC)*_*start*_ was observed during the AST against ciprofloxacin in the samples pre-exposed with 0.25X MIC and 0.5X MIC of ciprofloxacin, with around 24-fold decrease in (*iMIC)*_*start*_ as compared to control (unexposed) sample.

For the ASTs against streptomycin, pre-exposure with 0.5X MIC ciprofloxacin, decreased (*iMIC)*_*start*_ value by 17-fold to 0.03 µg/ml, in comparison to 0.5 µg/ml in control (unexposed) sample. Interestingly, 0.67 µg/ml, the only elevated (*iMIC)*_*start*_ value with respect to control (unexposed) sample, was seen in 0.125X MIC ciprofloxacin pre-exposure.

In our study, we used *(iMIC)*_*all*_ as a clear indicator of resistance of a section of population against high antibiotic concentrations. The higher the *(iMIC)*_*all*_ value, the greater the ability of bacteria to survive in antibiotic environment. During the ASTs against ciprofloxacin, the noteworthy increase was in 0.25X MIC of streptomycin pre-exposure, where *(iMIC)*_*all*_ was 18 ng/ml as compared to 15 ng/ml of the control (unexposed) sample.

Similarly for the ASTs against streptomycin, (*iMIC)*_*all*_ with 0.125X MIC of ciprofloxacin pre-exposure was found to be highest i.e. 1.93 µg/ml, in comparison with 1.55 µg/ml of the control (unexposed) sample. Here, the lowest value, 1.36 µg/ml, of (*iMIC)*_*all*_ was seen in the sample pre-exposed with 0.25X MIC of streptomycin.

In the pre-exposure experiments with 0.25X MIC and 0.5X MIC of ciprofloxacin for AST against ciprofloxacin because of unpredictable bacterial growth response and due to the nature of the Gompertz function, it was impossible to determine (*iMIC)*_*all*_. The Gompertz fit line was infinitely long, although parallel and very close to the concentration axis. In such cases the experimentally determined value, *(iMIC)*_*exp*_, was considered to be the concentration for complete growth inhibition, which is analogous to *(iMIC)*_*all*_ as both the values are numerically very close (Table 1; Figure 1).

**Table 1:** Parameters used in the paper to assess the heteroresistance profile after susceptibility testing with ciprofloxacin.

	Unexposed	Pre-exposure with ciprofloxacin			Pre-exposure with streptomycin		
		0.125X MIC	0.25X MIC	0.5X MIC	0.125X MIC	0.25X MIC	0.5X MIC
*(iMIC)*_*mode*_	9.2	9.9	2.4	2.2	9.5	10	9.5
*(iMIC)*_*mean*_	8.9	9.6	6.8	5.1	9.3	9.8	9.3
SD	2.3	4.3	5.3	3.7	2.7	3	2.6
RMSD	0.25	0.26	**2.20**	**1.68**	0.28	0.3	0.27
CoV	0.26	0.27	0.78	0.73	0.29	0.31	0.28
*(iMIC)*_*start*_	3.09	3.09	0.13	0.13	2.73	2.57	2.9
*(iMIC)*_*total*_	12.5	14.11	15.94	14.5	13.7	14.56	13.28
*(iMIC)*_*all*_	15	15.94	ND	ND	15.94	18	15.9
*(iMIC)*_*exp*_	14	16	16	16	16	18	16
DoH	4.86	5.16	**122.62**	**111.54**	5.84	7	5.48
Kurtosis	2.8 (± 3e-07)	2.76 (± 3.7-07)	5.43 (± 2.3e-05)	4.88 (± 7e-06)	2.74 (± 1.3e-08)	2.72 (± 1.7e-07)	2.76 (± 1.7e-06)
Skewness	-0.17 (± 9.7e-09)	-0.11 (± 7.3e-09)	**1.3** **(± 4.8e-07)**	**1.2** **(± 3.2e-10)**	-0.07 (± 4.3e-09)	-0.01 (± 3.2e-09)	-0.1 (± 6.2e-09)
p-value	-	0.648 (Insignificant)	1.026e-59 (Significant)	1.440e-76 (Significant)	0.892 (Insignificant)	0.677 (Insignificant)	0.857 (Insignificant)

SD: Standard deviation; RMSD: Root-mean square deviation; CoV: Coefficient of variance; ND: Not determinable. The units of concentrations, wherever applicable, are in ng/ml. Also, most important results are bolded. A t-test was performed between the control (unexposed) sample and individual pre-exposed samples, and the p-values < 0.05 are considered as statistically significant.

**Table 2:** Parameters used in the paper to assess the heteroresistance profile after susceptibility testing with streptomycin.

	Unexposed	Pre-exposure with ciprofloxacin			Pre-exposure with streptomycin		
		0.125X MIC	0.25X MIC	0.5X MIC	0.125X MIC	0.25X MIC	0.5X MIC
*(iMIC)*_*mode*_	1.1	1.5	0.74	0.33	1.1	0.93	0.88
*(iMIC)*_*mean*_	1.1	1.4	0.77	0.47	1.0	0.9	0.86
SD	0.2	0.25	0.31	0.28	0.27	0.19	0.25
RMSD	0.18	0.17	**0.42**	**0.85**	0.25	0.20	0.28
CoV	0.18	0.18	0.40	0.60	0.27	0.21	0.29
*(iMIC)*_*start*_	0.50	0.67	0.13	0.03	0.34	0.38	0.25
*(iMIC)*_*total*_	1.36	1.76	1.3	1	1.48	1.19	1.30
*(iMIC)*_*all*_	1.55	1.93	1.62	1.42	1.69	1.36	1.48
*(iMIC)*_*exp*_	2	2	1.5	2	2	2	1.5
DoH	3.1	2.88	**12.46**	**47.32**	4.97	3.58	5.92
Kurtosis	3.06 (± 8.4e-09)	3.13 (± 1.3e-09)	2.78 (± 6.8e-09)	3.65 (± 6.5e-09)	2.78 (± 6.5e-09)	2.96 (± 2.7e-09)	2.74 (± 1e-10)
Skewness	**-**0.39 (± 6.6e-09)	-0.43 (± 6.7e-09)	**0.27** **(± 1.3e-08)**	**0.82** **(± 4.4e-12)**	-0.14 (± 1.5e-10)	-0.32 (± 3.4e-09)	-0.07 (± 9.6e-11)
p-value	-	0.408 (Insignificant)	0.045 (Significant)	5.835e-13 (Significant)	0.848 (Insignificant)	0.591 (Insignificant)	0.354 (Insignificant)

SD: Standard deviation; RMSD: Root-mean square deviation; CoV: Coefficient of variance. The units of concentrations, wherever applicable, are in µg/ml. Most important results are bolded. A t-test was performed between the control (unexposed) sample and individual pre-exposed samples, and the p-values < 0.05 are considered as statistically significant.

**Fig. 2:**
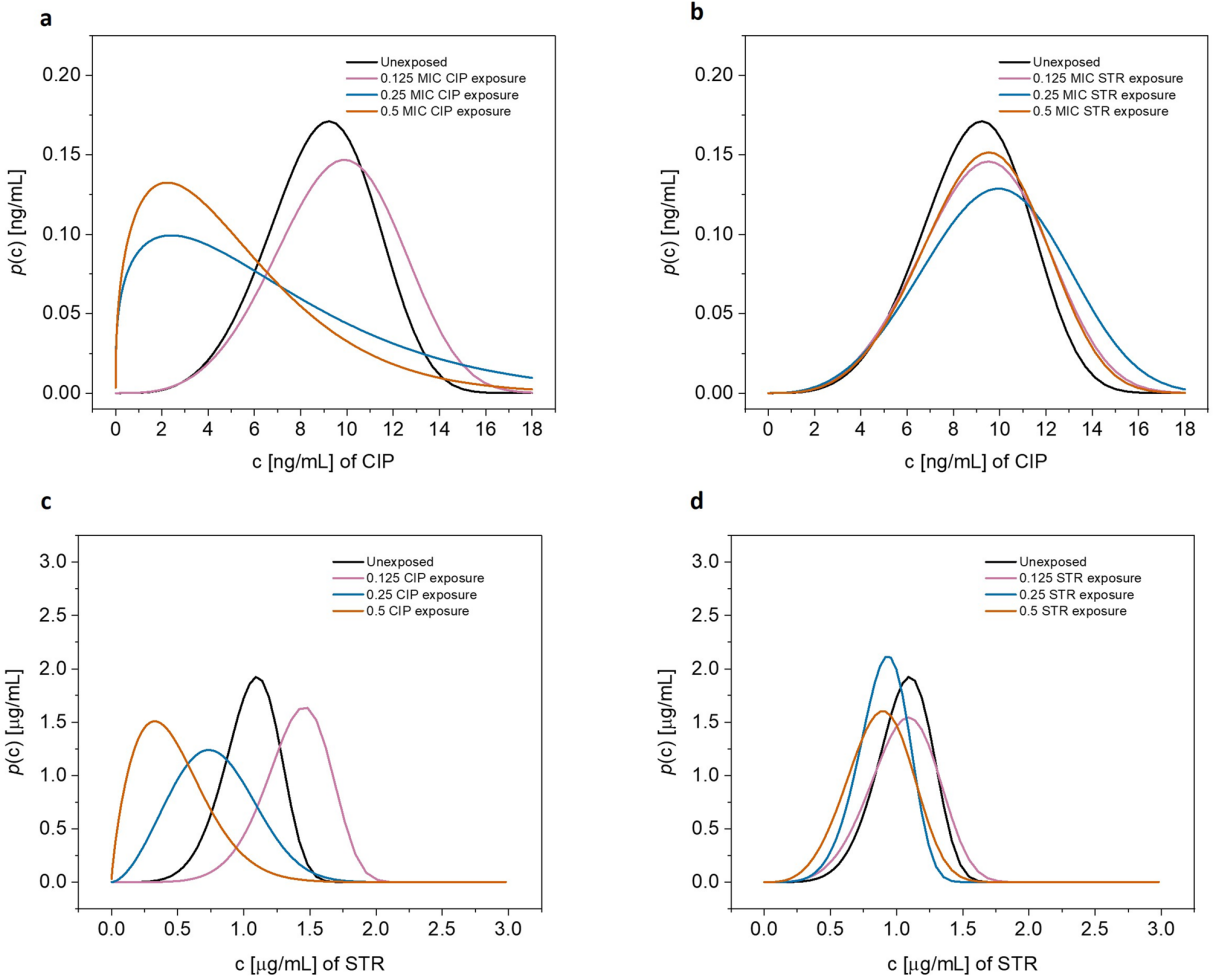
Probability distribution of *iMIC*. CIP: ciprofloxacin, STR: streptomycin. (**a**) and (**b**) represent the probability distributions of *iMIC* for susceptibility testing with ciprofloxacin in the cells pre-exposed with different independent sub-minimum inhibitory concentrations of ciprofloxacin and streptomycin respectively. Similarly, (**c**) and (**d**) show the probability distribution of *iMIC* for susceptibility against streptomycin in cells independently pre-exposed with different sub-minimum inhibitory concentrations of levels ciprofloxacin and streptomycin respectively. “Unexposed” in each panel refers to the control (unexposed) sample which was not pre-exposed. The width of each distribution is directly proportional to diversity in *iMIC* values and gives a rough idea about degree of heteroresistance. Skewness, which is the degree of asymmetry of the curve, is also visible as seen by the tailings on the left and right sides of the maximal point on each curve. Each curve is derived from a respective resistance profile; the latter was plotted first based on average values of three independent biological replicates (Figure F1; Supplementary figures SF2-SF4).

The width of *p(iMIC)* distribution curve gives a rough idea how heterogeneous the observed population of bacterial droplets is in terms of response to antibiotic. Greater the width of the curve on X-axis, greater the heteroresistance and vice versa (Figure 2). However, it is to be noted that this is just a visual and broadly superimposed depiction of all *p(iMIC)* distributions, we will standardize the heteroresistance deduction later in the manuscript for more accuracy.

## Effect on heteroresistance

An established parameter, degree of heteroresistance (DoH), which is the ratio between *(iMIC)*_*all*_ and *(iMIC)*_*start*_, is used to assess the *iMIC* distribution patterns and to make a direct comparison between all the experimental samples. DoH describes how spread the probability distribution of *iMIC* is between a specific antibiotic concentration range, with the distribution having greater spread implying a greater heteroresistance, in other words, the bacteria encapsulated droplets had greater diversity in the *iMIC* values.

**Fig. 3:**
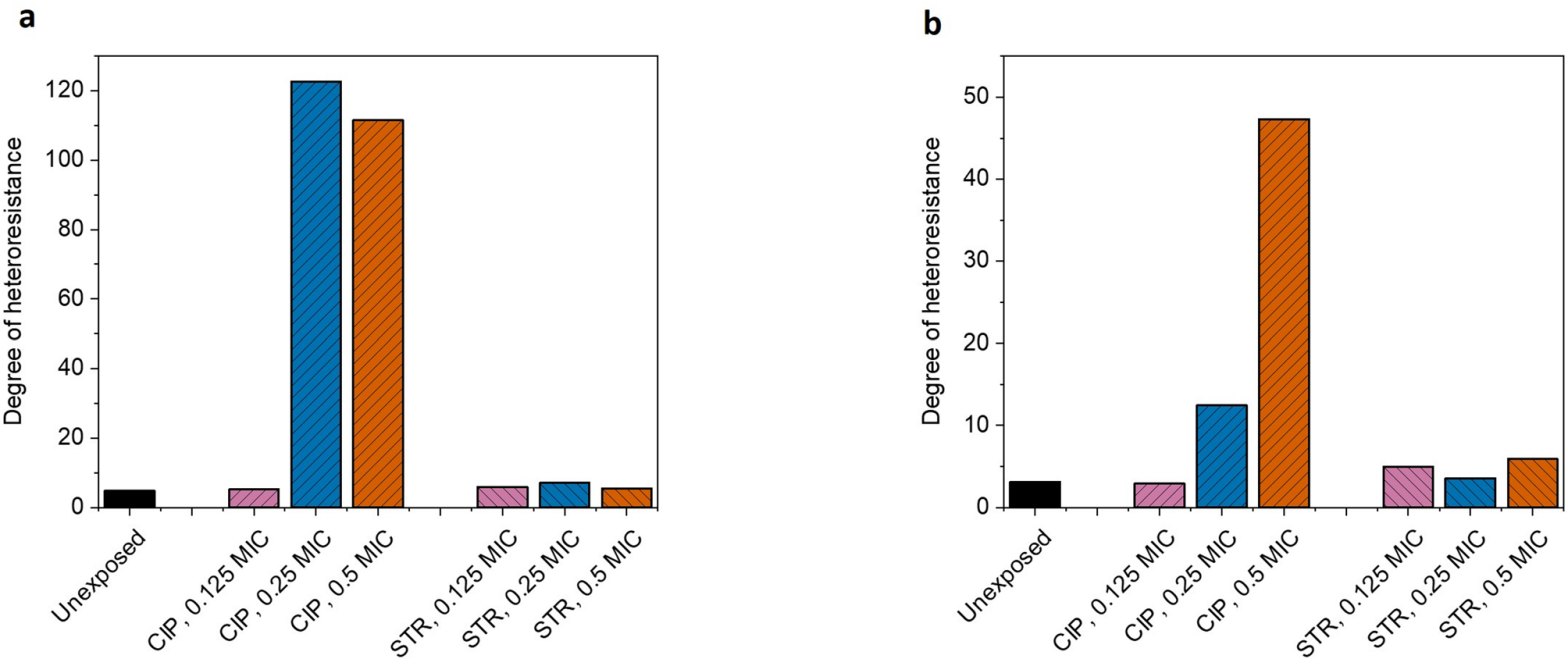
Graph showing the degree of heteroresistance (DoH). CIP: ciprofloxacin, STR: streptomycin. DoH is the ratio between *(iMIC)*_*all*_ and *(iMIC)*_*start*_, useful in quantifying the width of *iMIC* distribution on the concentration scale. These required parameters for DoH calculation were derived from Gompertz fitting, while the fitting itself was performed using the average values of three independent biological replicates. Therefore, the statistical errors in the above figures are irrelevant, also impossible to calculate, however, more information about replicates and statistics can be found in Figure 1; Supplementary figures SF2-SF4). (**a**) represents DoH observed during susceptibility testing with ciprofloxacin for bacterial populations independently pre-exposed with the different sub-minimum inhibitory concentrations of ciprofloxacin or streptomycin. A > 20-fold increase in DoH was observed in the population pre-exposed with 0.25X and 0.5X MIC of ciprofloxacin when compared with control (unexposed) sample. (**b**) shows DoH when susceptibility test was performed with streptomycin, independently, in the same pre-exposed cells. The sample pre-exposed with 0.5X MIC of ciprofloxacin showed 15-fold increase, hinting that the sub-MIC pre-exposure can influence the sensitivity of cells even to an antibiotic from another class which has a very different mechanism of action.

The analysis of the results showed a more than 20-fold increase in the degree of heteroresistance during AST against ciprofloxacin in the cells that were pre-exposed with 0.25X MIC and 0.5X MIC of ciprofloxacin, as compared to control (unexposed) sample (Figure 3).

Similarly, for the AST against streptomycin, 0.5X MIC ciprofloxacin pre-exposure topped the list, showing a 15-fold increase in the DoH, while the 0.25 MIC ciprofloxacin pre-exposure caused a 4-fold increase in comparison with the control (unexposed) sample, showing a cross-antibiotic effect on heteroresistance.

## Effect on skewness, standard deviation and root mean square deviation

In general, skewness (S) is defined as a measure for asymmetry of a curve, and here quantifies the degree of asymmetry in the *p(iMIC)* distribution. This gives an insight into whether the pre-exposure shifts the susceptibility distribution of individual cells towards concentrations greater or lower than *(iMIC)*_*mode*_ value.

**Fig. 4:**
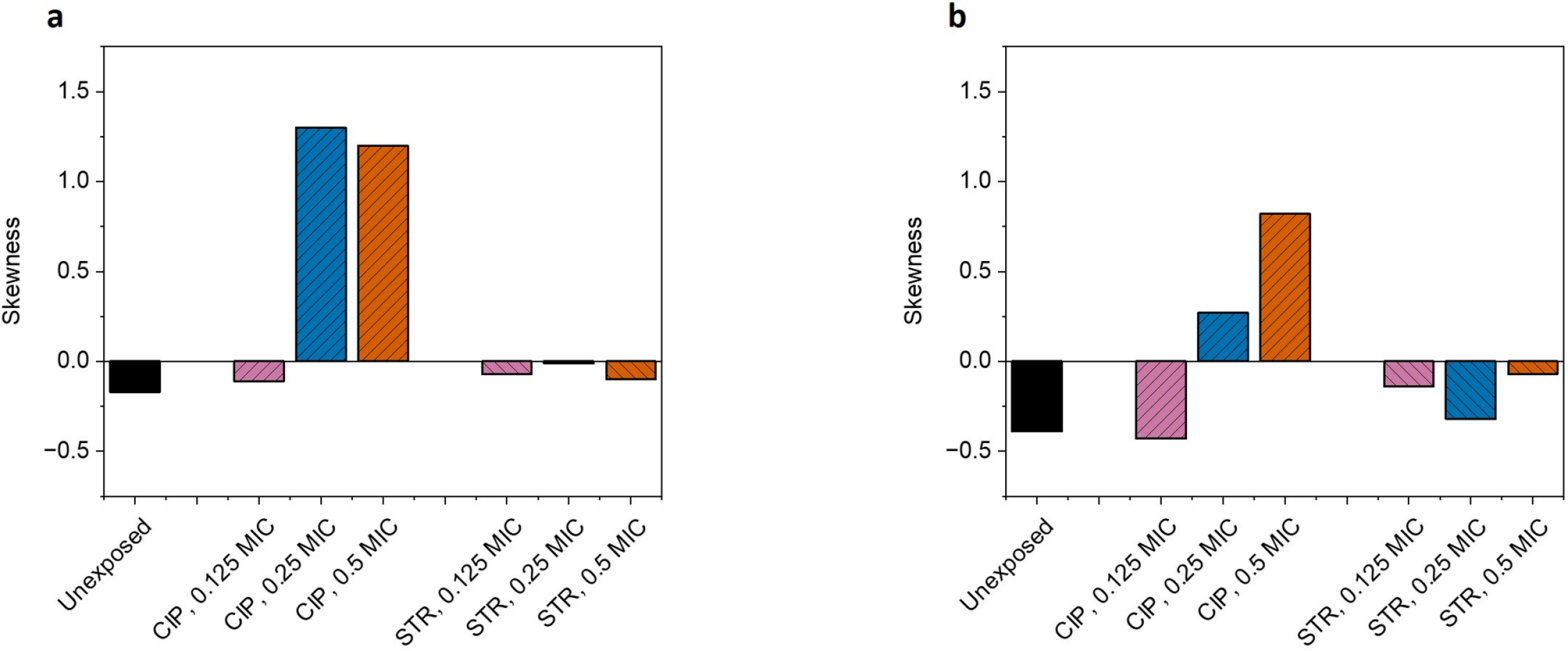
Plot representing skewness in *p(iMIC)* distribution. Skewness is a measure of asymmetry in the probability distribution, and refers degree of asymmetry in the *p(iMIC)* distribution. (**a**) represent skewness for AST with ciprofloxacin in bacterial populations independently pre-exposed with different sub-minimum inhibitory concentrations of ciprofloxacin or streptomycin. (**b**) shows the skewness when AST was performed with streptomycin, independently, in the cells pre-exposed with same concentrations of antibiotics. A positive skewness, as observed in pre-exposures with 0.25X MIC and 0.5X MIC ciprofloxacin, signifies that the bacterial response was more heterogeneous at concentrations greater than *(iMIC)*_*mode*_ than at concentrations lower than *(iMIC)*_*mode*_ value.

**Fig. 5:**
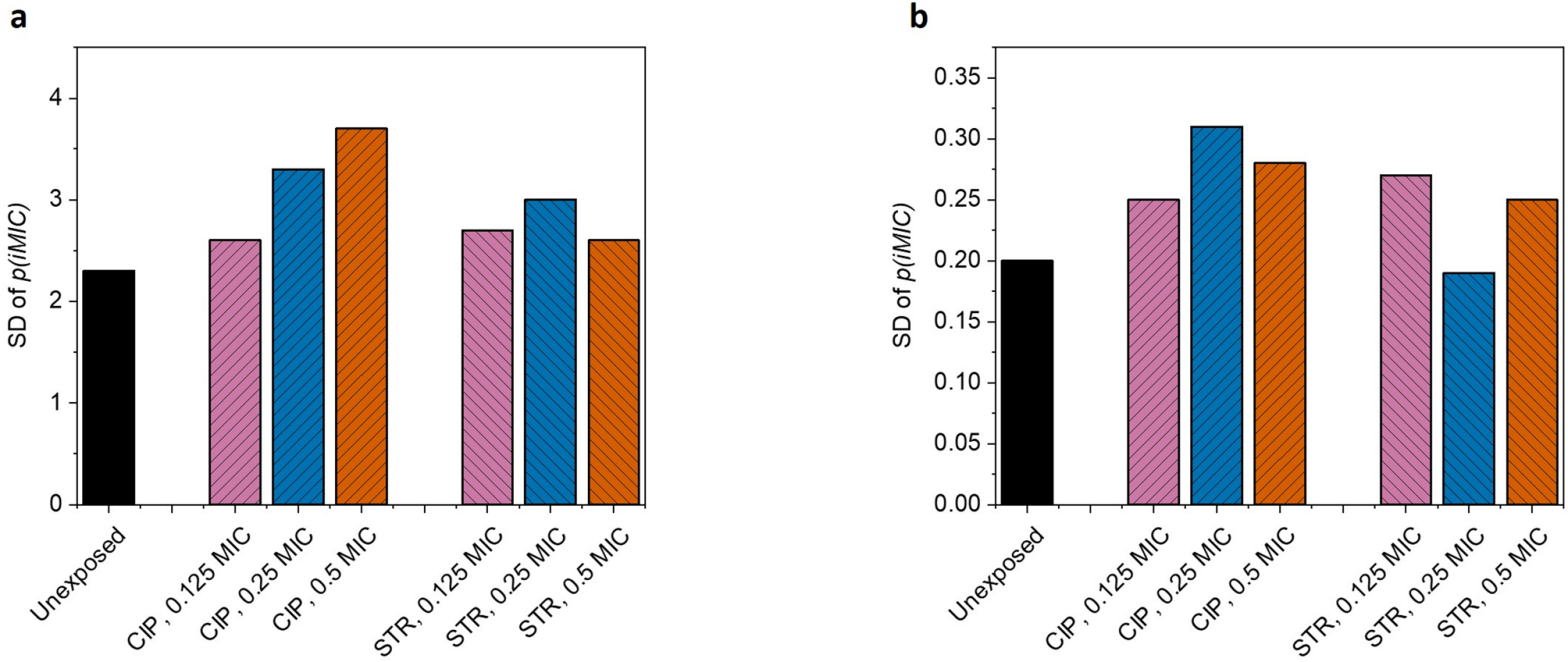
Plot representing standard deviation (SD) of *p(iMIC)*. (a) shows SD during AST with ciprofloxacin in bacterial populations that were independently pre-exposed with different sub-minimum inhibitory concentrations of ciprofloxacin or streptomycin. (**b**) shows the DoH when susceptibility test was performed with streptomycin after similar independent pre-exposures.

As evident from the observations (Table 1; Figure 4), during AST with ciprofloxacin, all pre-exposures showed negative skewness just like the control (unexposed) cells, except with 0.25X MIC and 0.5X MIC ciprofloxacin where skewness was + 1.3 and + 1.2 respectively. The above two pre-exposures also resulted positive skewness for susceptibility testing with streptomycin (Table 2; Figure 4), + 0.27 and + 0.82, with 0.125X MIC and 0.5X MIC ciprofloxacin respectively (Fig. 5).

For the *p(iMIC)* distribution during ASTs with ciprofloxacin, the standard deviation (SD) remained always high in all samples compared to control (unexposed) sample. A similar pattern was also observed during AST with streptomycin.

**Fig. 6:**
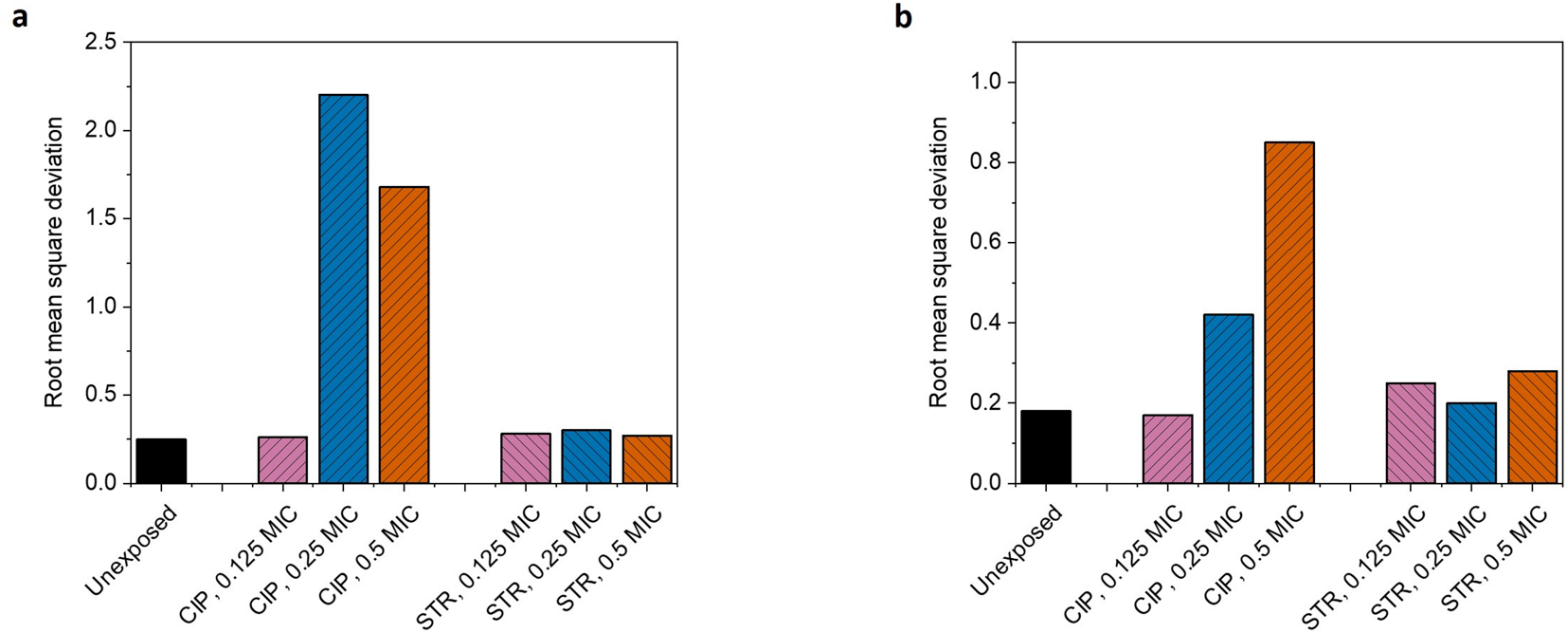
Plot representing root mean square deviation (RMSD). RMSD is the ratio between SD and *(iMIC)*_*mode*_ which is a more standardized measure of dispersion in the probability distribution and is used for unified comparisons. The parameters for RMSD calculation were derived from Gompertz fitting, and the fitting was performed using average of three independent biological replicates. Therefore, the statistical errors in RMSD are irrelevant, however, more information about replicates and statistics can be found in Figure 1; Supplementary figures SF2-SF4). (**a**) represent RMSD values for AST with ciprofloxacin in bacterial populations independently pre-exposed with different sub-minimum inhibitory concentrations of ciprofloxacin or streptomycin. (**b**) shows the RMSD values when susceptibility test was performed with streptomycin, independently, in the cells pre-exposed with same concentrations of antibiotics. RMSD is a very good indicator of heteroresistance in the population and it coincides with DoH calculations in the study.

Since each exposure changed the sensitivity of individual cells and their *iMIC* distributions to different extents, we used root mean square deviation (RMSD), a more standardized measure of dispersion to unify the comparison. Samples exposed with 0.25X MIC and 0.5X MIC of ciprofloxacin showed highest RMSD values in *p(iMIC)* distribution during AST against ciprofloxacin. These two pre-exposure concentrations also gave a similar pattern of *p(iMIC)* distribution during streptomycin AST (Figure 6). The analyzed results show that the pre-exposed samples showing higher RMSD also showed higher DoH.

## Discussion

Studying antibiotic resistance at the single-cell level is critically important for the fact that it provides insights into individual cell response which cannot be achieved from bulk population studies. Even in genetically identical bacterial populations, some cells may survive the antibiotic treatment due to transient phenotypic states^34^. Importantly, just a single surviving bacterium can have the ability to proliferate into a population and cause a future infection^43^. Identifying and understanding these surviving sub-populations and the total heteroresistance of the population in general, can improve strategies for the development of more effective antibiotics, treatment regimens and diagnostic tools, ultimately helping to combat the global antibiotic resistance crisis. Although our approach with droplet microfluidics is just binary and cannot capture the response of metabolically inactive cells, it was still informative in the aspect of determining *iMIC* and elucidating the heteroresistance of cells in the population^44^.

The transient phenotypic state, mentioned above is a temporary state in which bacterial cells show resistance to an antibiotic without acquiring genetic mutations. This can be generally induced by exposure to antibiotics, even at low levels, stressing the bacterial cells and inducing adaptive, reversible changes in phenotype. Temporary changes can include alterations in drug uptake and efflux, or modifications to antibiotic target sites. These states can typically arise in agriculture land, industrial discharge and hospital waste due to improper antibiotic use^14,37,45^. Bacteria can get exposed to the residual antibiotics in the environment itself or after the antibiotic enters animals or humans, typically through oral route. The continuous sub-lethal level antibiotic exposure in general can act as a stepping stone for genetic resistance, lead to treatment failure, create complexity in resistance surveillance and thereby impacting public as well as environmental health.

Previously, many studies have examined the antibiotic-resistant mutant selection at antibiotic concentrations greater than MIC, but limited resources are available about the effects of sub-MIC exposures^25,46,47^. Moreover, most of these studies were bulk population observations^48–50^ and the response of individual cells was not considered. Keeping this in mind, to elucidate single-cell response on short-term exposures, ciprofloxacin- a fluoroquinolone antibiotic, known to induce ROS formation^11,51^promote DNA mutations^52^ and cause evolution of high-level resistance^51,53^ was chosen for the current study. Another antibiotic, streptomycin, an aminoglycoside also known to promote DNA mutations^54^ and cause high resistance selection^55^ was also studied in the same manner.

Differences in bacterial cells within a population exist in the context of cell differentiation and division, formation of spores, activity of enzymes, stress responses^21^. The genetic, epigenetic, and non-genetic mechanisms can be the underlying reasons for these variations. On top of the existing heterogeneity in the population, the sub-MIC antibiotic exposure the damage to DNA, protein and membrane lipids may invoke oxidative stress response or SOS response^9,56^leading to heterogeneous physiological, metabolic and morphological changes, thereby presenting diverse response to subsequent antibiotic exposures. The stress response pathways can activate protective mechanisms such as DNA repair, protein chaperons and reduce metabolic activity, which help bacteria tolerate the antibiotic better. The pre-exposure can also promote the formation of dormant or slow growing variants, persisters^25^lead to activation of efflux pumps, cause alteration of antibiotic target, thereby, altering heteroresistance of the population.

During our classic MIC testing using the broth dilution method, we recorded the growth kinetics at different antibiotic concentrations by measuring the optical density (600 nm) of culture. The MIC was then established based on concentration that inhibited the growth (Supplementary figure SF1). The MICs of ciprofloxacin and streptomycin were found to be 12 ng/ml and 3 µg/ml respectively, while using the Gompertz fitting, the (*iMIC)*_*all*_ was calculated to be 15 ng/ml and 1.55 µg/ml for ciprofloxacin and streptomycin respectively.

For all the pre-exposure with ciprofloxacin, elevated (*iMIC)*_*all*_ values can be attributed to the same reasons as mentioned above i.e. ROS generation, DNA mutations and resistance selections^11,51–53^. However, 0.5X MIC of ciprofloxacin pre-exposure could have been too damaging to the cells that (*iMIC)*_*all*_ decreased to 1.42 µg/ml on subsequent streptomycin exposure. The highest (*iMIC)*_*all*_ value for ciprofloxacin sensitivity was found to be 18 ng/ml, in the pre-exposure with the 0.25X MIC streptomycin, however, specific reason for this elevation in *(iMIC)*_*all*_ unclear.

A comparison of *iMIC*_*all*_ values can also be made with MICs calculated in bulk. Generally, the results within a difference of one two-fold dilution are considered to be in “essential agreement” - especially considering that the two experiments were made with the use of two very different methods (broth dilution and droplets). We see that the MICs for both the antibiotics in bulk for all pre-exposed samples are same as the control (unexposed) sample. However, changed *iMIC*_*all*_ value for a sample with its respective bulk MIC can be attributed to greater sensitivity of single-cell detection method or due to concentration breakpoints used for broth dilution and droplet method. This also indirectly highlights the need to perform single cells studies for susceptibility testing. Moreover, our study objectives were not to compare difference in MIC between bulk culture or single cell, and for the same reason we did not dive deeper in any comparable differences observed.

As mentioned earlier, *(iMIC)*_*mode*_ is the most frequently occurring *iMIC* value for during single-cell AST. An increase in *(iMIC)*_*mode*_ value after pre-exposure implies that the majority of the population has evolved some mechanism to counter the antibiotic effect. The 0.5X MIC of ciprofloxacin pre-exposure considerably reduced the *(iMIC)*_*mode*_ during subsequent exposures to both ciprofloxacin and streptomycin, owing to residual inhibitory effects of ciprofloxacin, primarily toxic oxygen species. Ciprofloxacin is known to show synergistic effects with aminoglycosides as ciprofloxacin induced DNA damage can increase bacterial stress and vulnerability, enhancing the action of protein synthesis inhibitor antibiotics^53,57^. However, very low levels of ciprofloxacin pre-exposure imparted protective ability to most cells during re-exposures, even against streptomycin.

When analyzing the Gompertz fittings (Figure 1; Supplementary figures SF2-SF4), the bacterial population that was proliferating between the concentration at which first decrease in growth is observed, (*iMIC)*_*start*_, and the lowest concentration that completely inhibited growth of all cells, (*iMIC)*_*all*_, is most vital, as the ratio between latter to former is used to calculate the DoH. Before proceeding further in this discussion, noteworthy observation is that the higher concentrations of ciprofloxacin pre-exposures (0.25X MIC and 0.5X MIC) reduced the *(iMIC)*_*start*_ value (Table 1), hinting that re-exposure showed synergistic effect with the already damaging high initial antibiotic pre-exposure concentration, at least for a sizeable population. However, due to the heterogeneity, some cells still well tolerated higher levels of pre-exposure and subsequently became less sensitive to further antibiotic concentrations. Cumulatively, the heterogenous response of cells to pre-exposure and to subsequent exposure shaped the overall heteroresistance, as seen in pre-exposures with 0.25X MIC and 0.5X MIC ciprofloxacin (Figure 3). The synergistic (reduction of *(iMIC)*_*start*_ value) and the antagonistic behavior (promotion of heteroresistance) with ciprofloxacin pre-exposure is way more evident in the results than that with streptomycin pre-exposures.

We hypothesize that the ROS generation and DNA mutagenic potential behavior of ciprofloxacin is a stronger driving force for generating heteroresistance than translation inhibition caused by streptomycin. Our results were in agreement, at least indirectly, with a previous study^58^ which showed that protein synthesis inhibitor antibiotics cause less population growth rate heterogeneity than DNA synthesis inhibitor antibiotics. Moreover, the likelihood of increased heteroresistance and its degree is higher during the continued exposures with ciprofloxacin i.e. ciprofloxacin pre-exposure followed the AST against ciprofloxacin as compared to AST against streptomycin.

In our observations, we also found some correlations between the degree of heteroresistance and skewness, positive skewness was associated with higher degree of heteroresistance and vice versa (Tables 1 and 2). Negative skewness indicates that the bacterial response stayed more heterogenous at concentrations below *(iMIC)*_*mode*_ and the tailing is greater on the left side of the curve. While positive skewness with tailing on the right side of the curve points to higher heterogeneity in response on the right side, also indicating the presence of small sub-populations of bacteria that are more resistant than the majority of cells. Pre-exposures with 0.25X MIC and 0.5X MIC ciprofloxacin resulted in positive skewness for re-exposures with ciprofloxacin and streptomycin both, again strengthening the hypothesis that these two concentrations of ciprofloxacin pre-exposure have higher potential to create resistant variants of MG1655 than pre-exposures with streptomycin. Additionally, the potential of ciprofloxacin pre-exposure to create resistant variants against streptomycin is dose dependent, at least in our study, which is evident from more positive skewness in 0.5X MIC ciprofloxacin pre-exposure.

The type of antibiotic as well as the dose of pre-exposure is a crucial factor in generating heterogeneity at the sub-MIC level, as previously published^58^ results, where antibiotic exposure concentrations closer to MIC resulted in higher growth rate heterogeneity. In our study, the cross-antibiotic effect of ciprofloxacin pre-exposure influencing the susceptibility of the cells against streptomycin is an interesting observation. The exact mechanism for this remains unknown, but previous studies have mentioned antibiotic pairs showing cross-resistance or cross-sensitivity^59–61^. This can be inferred to the increased activity of broad-spectrum efflux pump or decreased uptake of antibiotic due to reduced membrane permeability. While direct data for bacterial response to streptomycin, postciprofloxacin exposure is limited, the trend observed with other antibiotics strongly suggests similar patterns. Further experiments with many different combinations of antibiotics at wide range of concentrations and various durations of pre-exposure along with genetic approaches can establish the antibiotics that are the most potent stimulants of heteroresistance.

A limitation during single-cell phenotypic AST with droplet microfluidics is the method’s inability to capture the behavior of cells which are either metabolically inactive or very slow growing in the presence of antibiotic. These cells cannot be distinguished from the dead cells, leading to false negative results, ultimately implicating the *iMIC* and DoH values. If these cells, after reverting to a normally growing phenotype, display an *iMIC* value greater than the majority of the population, it will impact the *iMIC*_*all*_ calculation. Moreover, based on the actual susceptibility of these cells, the overall probability density distribution of *iMIC* will change and so will the DoH. However, for our current study, we hypothesized that the fraction of such cells is negligible and single-cell phenotypic AST presents much more information than phenotypic AST methods in bulk culture.

## Conclusion

In this study of sub-MIC pre-exposure, we showed that ciprofloxacin can significantly increase the heteroresistance of bacterial population, not only against its subsequent exposure but also to that of streptomycin. Although this was a short-term pre-exposure study, we hypothesize that this phenotypic heterogeneity was generated due to ciprofloxacin’s ability to cause generation of ROS, long-term pre-exposure can lead to the selection of more resistant variants at sub-MIC levels^55,62^. The consistent sub-MIC level presence of antibiotics in the environment can create mild selection pressure and lead to the evolution of resistance. Not to forget, the misuse of antibiotics both at the end of clinical staff and patient brings the same problem in the clinical setup^37^.

Our results also emphasize the need of single-cell behavior elucidation as population studies in bulk fail to represent the whole picture of bacterial response to antibiotic and even a single surviving bacterium can be capable of establishing an infection at later stages. The data presented here offers a new perspective for the efficient antibiotic use to prevent genetic mutations and development of resistance. Furthermore, the technique of droplet microfluidics, we used in this study is not just limited to AST but can be also used in many different other domains of antibiotic resistance-based studies.

## Materials and methods

### Strains, media, antibiotics

All the sterilizations were performed in Varioklav steam sterilizer (HP Medizintechnik GmbH, Oberschleissheim, Germany) wherever required. The *Escherichia coli* MG1655 laboratory strain was used for all the experiments. The strain was directly streaked from glycerol stock on Mueller Hinton (MH) Agar plate and all the liquid culture were set in MH broth (DifcoTM, Becton, Dickinson and Company, MD, USA). Ciprofloxacin (TOKU-E, Japan) and Streptomycin (Alfa Aesar, USA) stock solutions were prepared in distilled water and further dilutions were freshly made in MH. All the reagents and growth media involving droplet generation or imaging was filtered with a 0.22 μm syringe filter (Googlab Scientific Sp. z o.o, Poland).

### Determination of minimum inhibitory concentration (MIC)

Appropriate antibiotic concentration range for MIC determination was chosen and 100 µl of each concentration was added in triplicates to a 96 well plate. Three isolated colonies were picked from freshly streaked agar plate to ensure sufficient heterogeneity, resuspended completely in MH and OD_600_ was taken using DiluPhotometer™ (Implen GmbH, München, Germany) and diluted to required concentration. 100 µl of diluted culture were seeded in all the wells containing antibiotics, to have 200 µl total volume with final bacterial concentration of 5 × 10^5^ CFU/ml and needed antibiotic concentrations. Positive, negative and media controls were also set. The plate was incubated at 37 °C for 18 h and the lowest concentration showing no visible growth was taken as MIC.

### Design and fabrication of microfluidic devices

For the droplet generator master chip, a CNC machine (MSG4025, Ergwind, Poland) was used to mill the pre-designed channel patterns having a flow focusing geometry on a polycarbonate (PC) plate (Macroclear, Bayer, Germany). To prepare polydimethylsiloxane (PDMS) mold, a 1:10 ratio of curing agent and PDMS (Sylgard 184, Dow Corning, USA) was thoroughly mixed, poured into a small casting tray with the PC chip and cured at a temperature of 75 °C for 2.5 h. The PC chip was carefully removed, and cleaned mold surface was silanized in a chamber with vapors of tridecafluoro-1,1,2,2-tetrahydrooctyl-1-trichlorosilane (United Chemical Technologies, USA) for 1 h under 15 mbar pressure. Freshly prepared PDMS and curing agent mixture was poured in the mold and cured as previously, followed by cutting out the chips from the mold. The PDMS chip was then plasma bonded to a 1 mm glass slide after using a plasma cleaner (Harrick Plasma, USA). To make the channels hydrophobic, Novec 1720 (3 M, USA) was gently flushed into microchannels and allowed to evaporate, repeating it for a total of three times (Supplementary figure SF6).

The imaging chip was fabricated by standard photolithography on a 4-inch Silicon wafer. The AutoCAD (2020, Version 1.4) was used to prepare the design and geometry of the photomask. A negative photoresist SU-8 (Kayaku Advanced Materials, USA) was spin coated at 3000 rpm to for desired channel specifications. Further preparation of PDMS chip as per standard methods. The spin coated wafer was then baked at 65 °C and 95 °C according to the specifications of the manufacturer. The Silicon master mold was exposed to UV, and processed for further development. PDMS was poured onto the developed master mold and cured in oven to obtain PDMS chips followed by bonding and channel modification for hydrophobicity, similar to the chip for droplet generation. 508 (Supplementary figure SF6).

### Pre-exposure

A single colony was picked up with a sterile loop and inoculated in a falcon with 10 ml MH, incubated overnight at 37 °C and 225 rpm. The culture next day was diluted 1: 100 in MH to a total of 10 ml. OD_600_ was monitored every 30 min to obtain an optical density of 0.1–0.2. The antibiotic for pre-exposure at desired concentration was added to MH in a conical flask, with 5 × 10^5^ CFU/ml final concentration of bacteria in it and a total volume of 20 ml. The incubation was done as earlier and OD_600_ recorded every 30 min till 0.1–0.2, at which the cells were harvested by centrifugation for 5 minutes at 4400 x g using a table top centrifuge (MPW Med. Instruments, Poland). The pellet was then resuspended in fresh MH media, followed by centrifugation pelleting again, repeating it for a total of three times to get rid of any residual antibiotics. After the final resuspension, the cells were ready for AST with ciprofloxacin and streptomycin.

For single-cell AST: A range of antibiotic concentration was prepared in a 96 well plate, followed by addition of pre-exposed bacteria, to get a final concentration of 1 × 10^5^ CFU/ml in every well. Poisson distribution was taken into consideration while deciding inoculum density, to ensure just one bacterium is encapsulated per droplet.

For bulk culture AST: A range of appropriate antibiotic concentrations were prepared in the 96-well plate and 100 µl of each concentration was added in triplicates. The pre-exposed bacteria were diluted to 1 × 10^6^ CFU/ml, and 100 µl of the culture was seeded in respective wells of the 96-well plate to bring the final required antibiotic concentration and 5 × 10^5^ CFU/ml bacterial concentration. The plate was incubated at 37 °C for 18 h and the lowest concentration showing no visible growth was taken as MIC.

### Microfluidic droplet generation

Novec 7500 fluorocarbon oil (3 M, USA) containing 2% FluoSurf-O™ (Emusleo, France) was used for making 1 nl droplet. The flow rate of oil and sample was optimized for required droplet size and controlled using syringe pumps NemesyS (Cetoni GmBH, Germany) and operated by the software QmixElements (Cetoni GmBH, Germany). Generated droplets were carefully collected in 0.2 µl microtubes and incubated at 37 °C for 18 h.

### Image acquisition

The droplets after incubation were taken up in a polytetrafluoroethylene tubing (Adtech SC, Poland) using the same setup as droplet generation and pushed in the image acquisition chip. The bright field images were acquired using an inverted microscope (Nikon Eclipse Ti2), equipped with a camera (Andor Zyla sCMOS) and 10X/0.30 objective (Nikon Plan Flour). Images were then extracted to 16-bit grey scale using the NIS-elements AR software (v5.41.00) for further analysis.

### Droplet analysis

A computer program was developed to analyze the droplet images previously acquired under bright field. This software was crafted in Python (v3.11.8) programming language, utilizing publicly accessible libraries such as Scikit-image and OpenCV to analyze the droplet images^63–65^.

A novel approach involving the analysis of pixel texture (Supplementary figure SF6-SF7) through grey-level co-occurrence matrices (GLCMs) of the droplets was used, as applied in aerial photography for Earth surface analysis^66^. The code first identifies individual droplets in the image using OpenCV methods, selects small patches and calculates GLCM properties for each patch. A plot is then generated showing correlation between each patch’s GLCM properties (dissimilarity, homogeneity), the center point for each droplet data (average on the axes) is determined and the distribution of these points for all droplets is displayed on a chart. Subsequently, a k-means clustering algorithm using the Sklearn is implemented and the grouping for droplets with or without bacteria can be done using the graphical overview.

To evaluate the effectiveness of the droplet image analysis method, bacteria expressing a fluorescent protein were used. After encapsulating the bacteria in droplets and incubating them, images of the droplet array were taken using a fluorescence microscope. The images were captured both under visible light and under fluorescence illumination. Using the developed analysis method, the droplets containing bacteria were identified based on the images taken under visible light. The detected positions of bacteria-containing droplets were then overlaid onto the image captured under fluorescence illumination. Analysis of this composite image demonstrated the effectiveness of the method. A total of 140 droplet images were analyzed, containing 15,858 droplets in total. Bacteria were detected in 1,447 (9.1%) droplets. There were 9 (0.6%) false negative results and 10 (0.7%) false positives (Supplementary figure SF8).

### Calculations of individual MIC and its probability distribution

After the image analysis, for the droplet library of every antibiotic concentration, the total number of droplets were enumerated, *N(c)*. The number of positive droplets i.e. having successful bacterial encapsulation and showing growth, *N*_*+*_*(c)*, were also counted.

The positive fraction of the droplets is denoted as *f*_*+*_*(c) = N*_*+*_*(c)/N(c)*. For each experimental set, to accurately analyze the growth of bacteria under antibiotics at every concentration, the number of positive droplets for each concentration, *f*_*+*_*(c)*, were normalized with the total number of positive droplets in the droplet library without any antibiotic, *f*_*+*_*(0).* This gave us the fraction of individual bacterial cells that were able to successfully proliferate as a function of antibiotic concentration: *F*_*R*_*(c) = f*_*+*_*(c)/f*_*+*_*(0).* The experimental data points for each fraction of individual cells that proliferate, *F*_*R*_*(c)*, for each antibiotic concentration were plotted against concentration and a fitting was done (Figure 1; Supplementary figure SF2-SF4) using Gompertz function^39,41,42^: $\:\phi\:\left(c\right)=exp{\{-(\frac{c}{{p}_{1}})}^{{p}_{2}}\}$, where *c* is the antibiotic concentration, *p*_*1*_ represents concentration at maximum slope, and *p*_*2*_ denotes slope parameter at *c = p*_*1*_. This fitting was done using the average values of *N(c)* and *N+(c)* obtained from three independent biological replicates.

Using the Gompertz function the inflection point of the curve was also calculated, which mathematically represents the second derivative of *F*_*R*_*(c)* equals zero for *c = c*_*(iMIC)*_.

After solving this equation *d*^*2*^*F*_*R*_*(c)/dc*^*2*^ *= 0* we get $\:{c}_{\left(iMIC\right)mode}={p}_{1}{\left\{1-\frac{1}{{p}_{2}}\right\}}^{\frac{1}{{p}_{2}}}$.

To calculate *p(iMIC)*, which is the probability density distribution of *iMIC* and represents the probability that the concentration will inhibit the growth of a bacterium, we utilized the expression *p(c) = - dF*_*R*_*(c)/dc.* This is derived from the Gompertz function *φ(c)*, and the calculation of probability distribution is done by:$\:p\left(c\right)=\frac{{p}_{2}{c}^{\left({p}_{2}-1\right)}}{{{p}_{1}}^{{p}_{2}}}exp{\{-(\frac{c}{{p}_{1}})}^{{p}_{2}}\}$. For the parameter *p(c)*, we calculated the average value µ, variance σ and skewness γ, utilizing the gamma function **Г**(x):$\:\:\mu\:={\int\:}_{0}^{\infty\:}dc\:c\:p\left(c\right)$, $\:{\sigma\:}^{2}=$ $\:{\int\:}_{0}^{\infty\:}dc\:{(c-\mu\:)}^{2}p\left(c\right)$ and $\:\gamma\:=\frac{{\mu\:}_{3}}{{\sigma\:}^{3}}\:$where $\:{\sigma\:}^{3}=$ $\:{\int\:}_{0}^{\infty\:}dc\:{(c-\mu\:)}^{3}p\left(c\right)$ and $\:{\mu\:}_{3}\:=$ $\:{\int\:}_{0}^{\infty\:}dc\:{(c-\mu\:)}^{3}p\left(c\right)$:

$$\:\mu\:=\left(\raisebox{1ex}{${p}_{1}$}\!\left/\:\!\raisebox{-1ex}{${p}_{2}$}\right.\right){\Gamma\:}\left(\raisebox{1ex}{$1$}\!\left/\:\!\raisebox{-1ex}{${p}_{2}$}\right.\right);\:\:\sigma\:=\sqrt{\left(\raisebox{1ex}{$2{p}_{1}^{2}$}\!\left/\:\!\raisebox{-1ex}{${p}_{2}$}\right.\right)\:{\Gamma\:}\left(\raisebox{1ex}{$2$}\!\left/\:\!\raisebox{-1ex}{${p}_{2}$}\right.\right)-\left(\raisebox{1ex}{${p}_{1}^{2}$}\!\left/\:\!\raisebox{-1ex}{${p}_{2}^{2}$}\right.\right){\:{\Gamma\:}}^{2}\:\left(\raisebox{1ex}{$1$}\!\left/\:\!\raisebox{-1ex}{${p}_{2}$}\right.\right)}\:;$$

$$\:{\upgamma\:}=\frac{\left[\left(\raisebox{1ex}{$3{p}_{1}^{3}$}\!\left/\:\!\raisebox{-1ex}{${p}_{2}$}\right.\right)\:{\Gamma\:}\left(\raisebox{1ex}{$3$}\!\left/\:\!\raisebox{-1ex}{${p}_{2}$}\right.\right)-\:\mu\:(3{\sigma\:}^{2}+\:{\mu\:}^{2})\right]}{{\sigma\:}^{3}}$$.

## Supplementary Information

Below is the link to the electronic supplementary material.

Supplementary Material 1

## Data Availability

Data for this article, including the code for droplet analysis, code used for the raw data calculations and results for raw data fitting into the Gompertz function, are available at RepOD at https://doi.org/10.18150/JMSVYS.
