# Supplementary material for: Ciprofloxacin pre-exposure influences individual cell MIC and heteroresistance of bacteria inside microfluidic droplets
Source: Sci Rep. 2025 Sep 25;15:32889. doi: 10.1038/s41598-025-17157-0 (PMC12464320; doi:10.1038/s41598-025-17157-0)
Supplement: Supplementary file 1 — Supplementary Material 1 [file 41598_2025_17157_MOESM1_ESM.pdf]

Supplementary table ST1

Antibiotic	MIC	0.5X MIC	0.25X MIC	0.125X MIC
Ciprofloxacin	12 ng/ml	6 ng/ml	3 ng/ml	1.5 ng/ml
Streptomycin	3 μg/ml	1.5 μg/ml	0.75 μg/ml	0.375 μg/ml

Supplementary table ST1: Minimum inhibitory concentrations and the sub-minimum inhibitory concentrations used: table lists the MICs of ciprofloxacin and streptomycin against *Escherichia coli* MG1655 as determined by broth dilution method. Information about various concentrations for the pre-exposure is also given.

Supplementary table ST2

		**Pre-exposure with CIP**			**Pre-exposure with STR**		
	Unexposed	***0.125X MIC***	***0.25X MIC***	***0.5X MIC***	***0.125X MIC***	***0.25X MIC***	***0.5X MIC***
MIC of CIP (ng/mL)	12	12	12	12	12	12	12
MIC of STR (μg/mL)	3	3	3	3	3	3	3

Supplementary table ST2: Minimum inhibitory concentrations for control (unexposed) and pre-exposed samples in bulk culture: table lists the MICs of ciprofloxacin and streptomycin against *Escherichia coli* MG1655 when pre-exposed with different antibiotic concentrations, as determined by broth dilution method.

Supplementary figure SF1

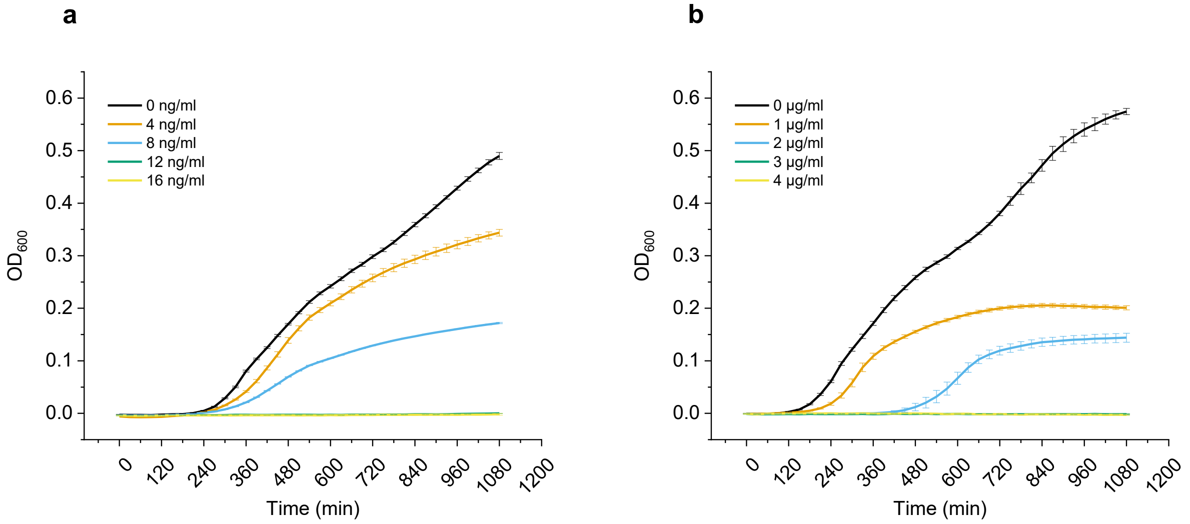

Supplementary figure SF1: The kinetics of MG1655 growth at different concentrations of antibiotic: (**a**) represents the growth at different concentrations of ciprofloxacin. (**b**) growth kinetics at different concentrations of streptomycin. Each data point is the average value of three independent biological replicates and the error bar represents the standard error. Black lines (0 ng/ml in (**a**) and 0 μg/ml in (**b**)) represent the bacterial growth kinetics without any antibiotic.

Supplementary figure SF2

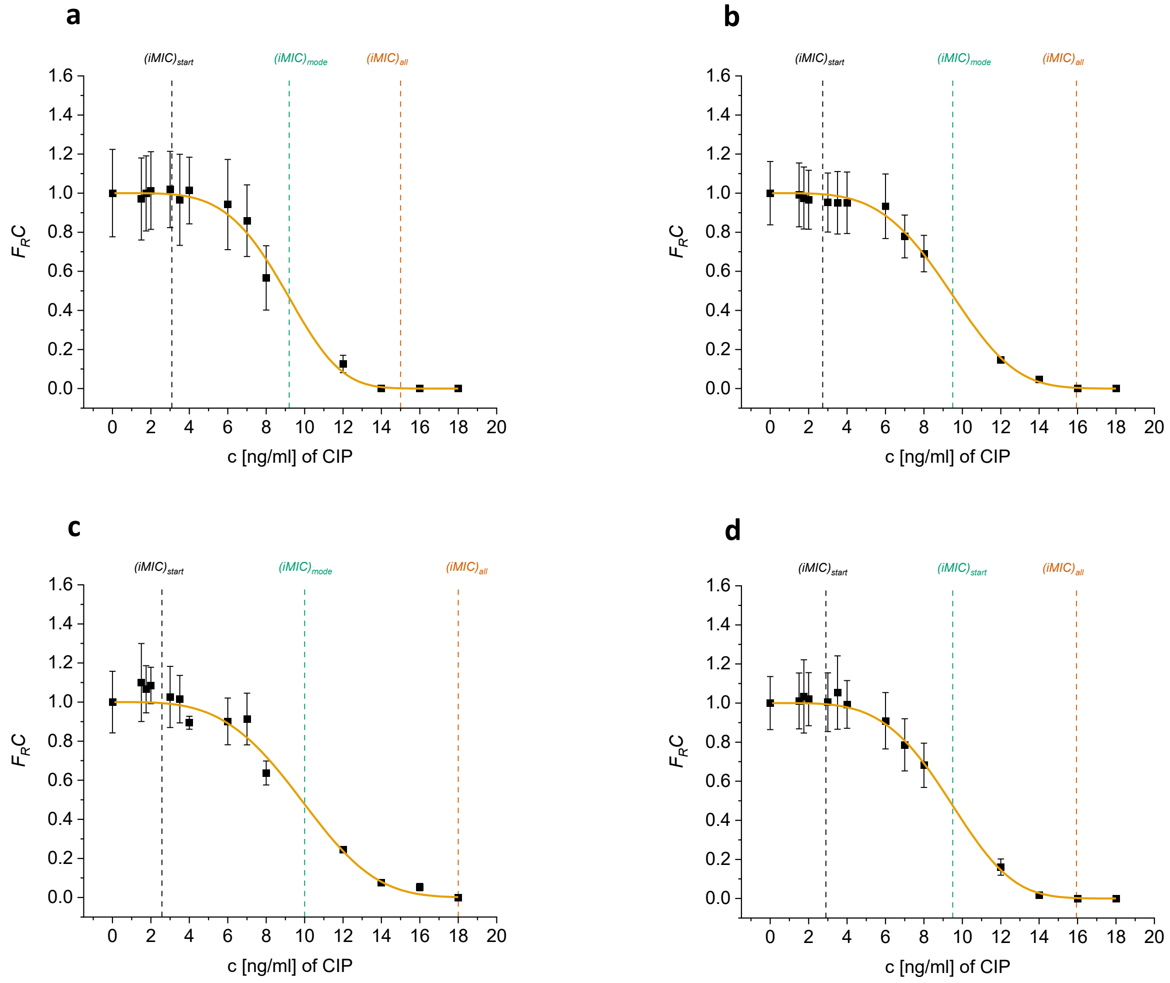

Supplementary figure SF2: plot shows the resistance profile and the Gompertz fit line for positive fraction of droplets, F_RC, as a function of antibiotic concentration, c, from lowest to highest. The susceptibility tests were performed independently against ciprofloxacin (CIP) on (**a**) control (unexposed) sample and (**b**) bacterial sample pre-exposed with streptomycin (STR) at a concentration of 0.125X MIC, (**c**) 0.25X MIC, (**d**) 0.5X MIC. Each black solid square data points are average of three independent biological replicates and error bars represent the standard deviations.

Supplementary figure SF3

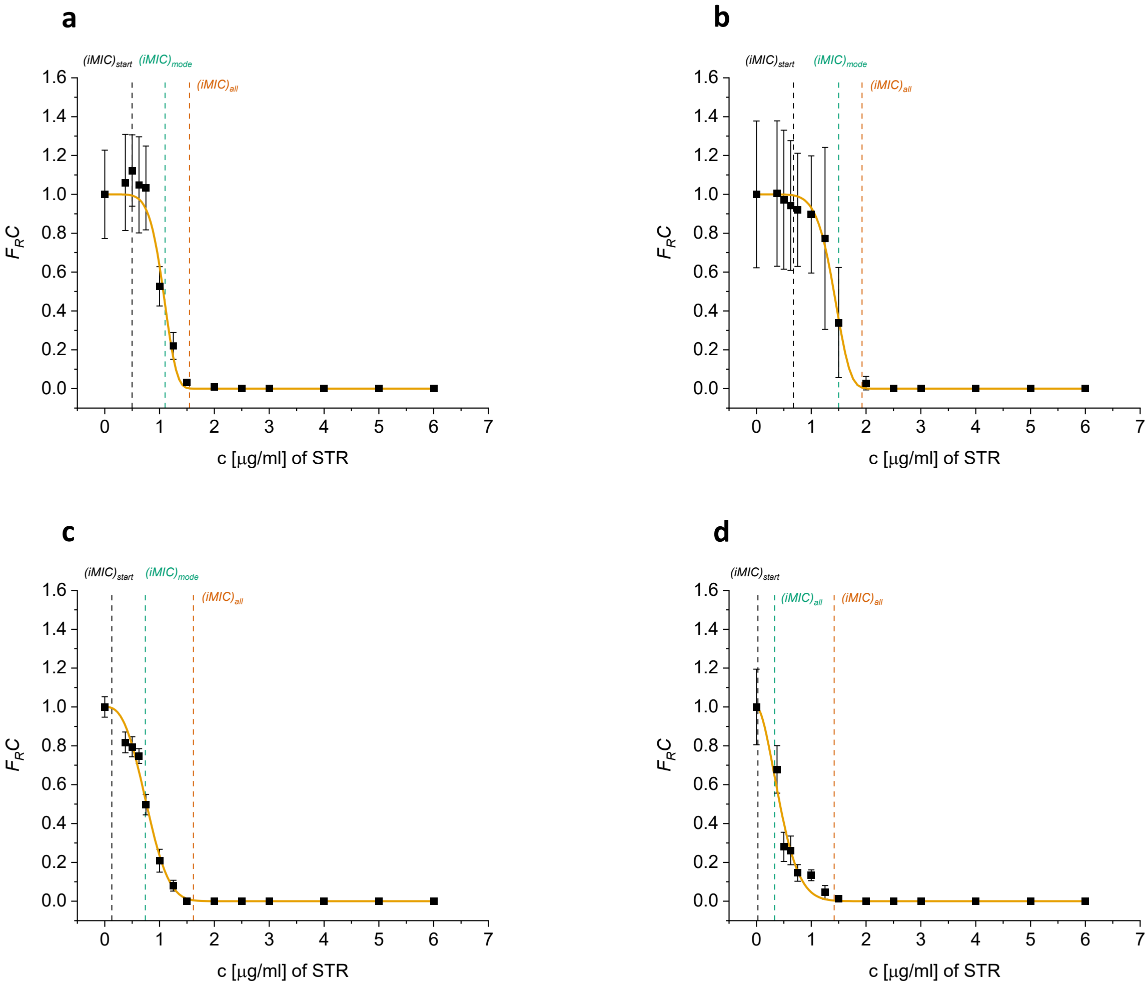

Supplementary figure SF3: graph shows the resistance profile and the Gompertz fit line for positive fraction of droplets, *$F_R C$*, as a function of antibiotic concentration, c, from lowest to highest. The susceptibility tests were performed independently against streptomycin (STR) on (**a**) control (unexposed) sample and (**b**) bacterial sample pre-exposed with ciprofloxacin (CIP) at 0.125X MIC, (**c**) 0.25X MIC, (**d**) 0.5X MIC levels. Each black solid square data points are average of three independent biological replicates and error bars represent the standard deviations.

Supplementary figure SF4

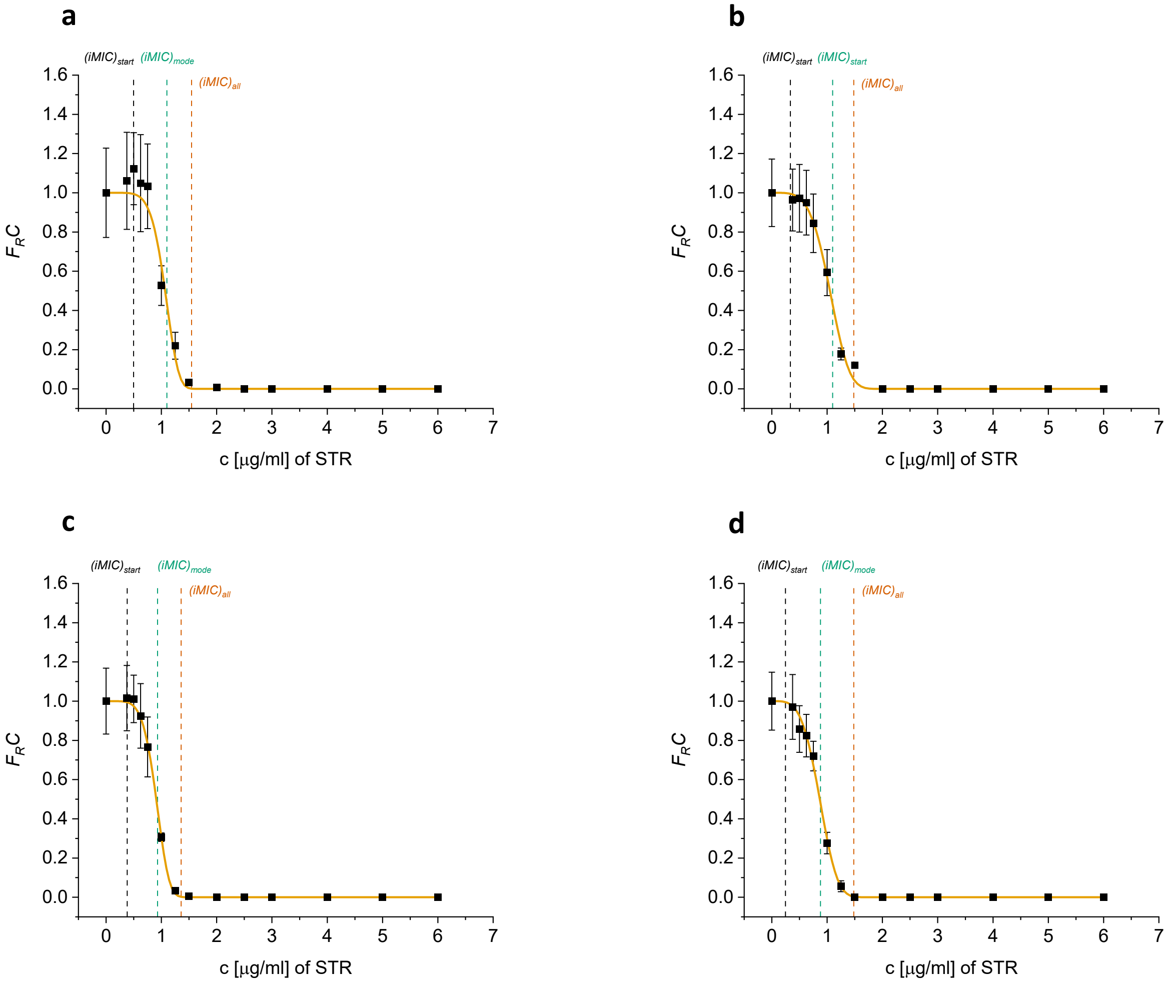

Supplementary figure SF4: plot depicts the resistance profile and the Gompertz fit line for positive fraction of droplets, F_RC, as a function of antibiotic concentration, c, from lowest to highest. The susceptibility tests were performed independently against streptomycin (STR) on the (**a**) control (unexposed) sample and (**b**) sample pre-exposed with 0.125X MIC of streptomycin, (**c**) 0.25X MIC of streptomycin, (**d**) 0.5X MIC of streptomycin. Each black solid square data points are average of three independent biological replicates and error bars represent the standard deviations.

Supplementary figure SF5

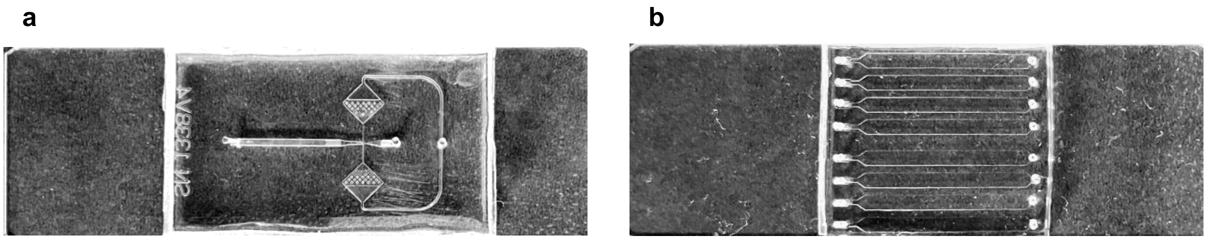

Supplementary figure SF5: shows pictures of microfluidic chips used in the study. (**a**) an image of droplet generation chip, (**b**) the picture of chip with chambers for droplet imaging.

Supplementary figure SF6

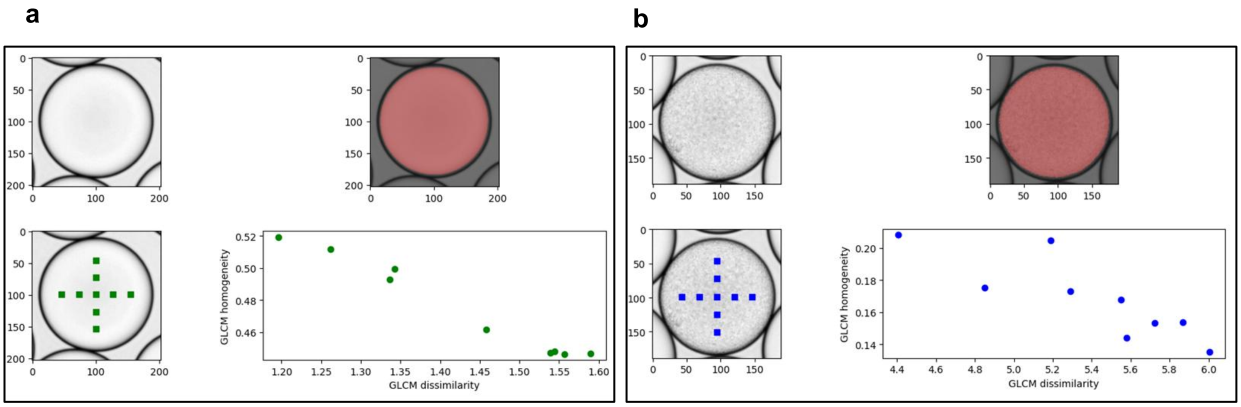

Supplementary figure SF6: depicts analysis of the pixel texture through grey-level co-occurrence matrices (GLCMs). (**a**) for an empty droplet, (**b**) for a droplet containing bacteria

Supplementary figure SF7

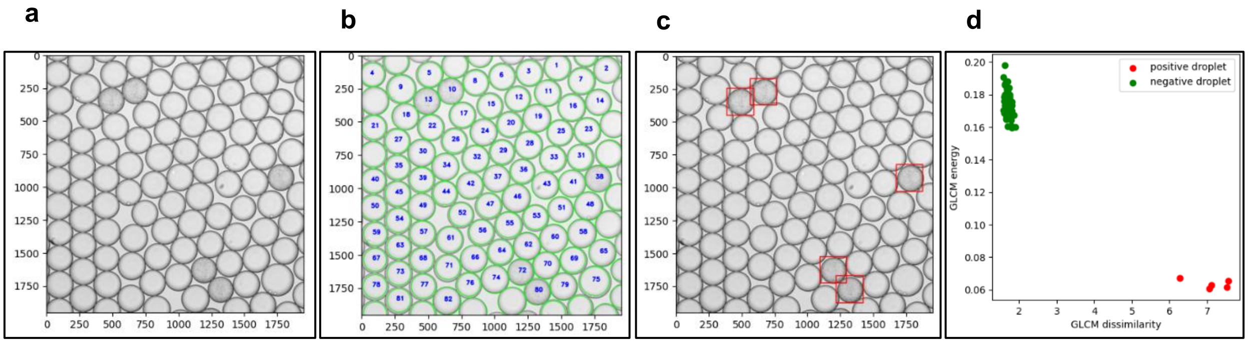

Supplementary figure SF7: shows the whole flow of droplet analysis from images. (**a**) single acquired image from the microscope, (**b**) identification of individual droplets and their enumeration, (**c**) identification of droplets containing bacteria and (**d**) grouping the droplets based on analysis of pixel texture GLCMs. *Note:* scales are not relevant in the pictures.

Supplementary figure SF8

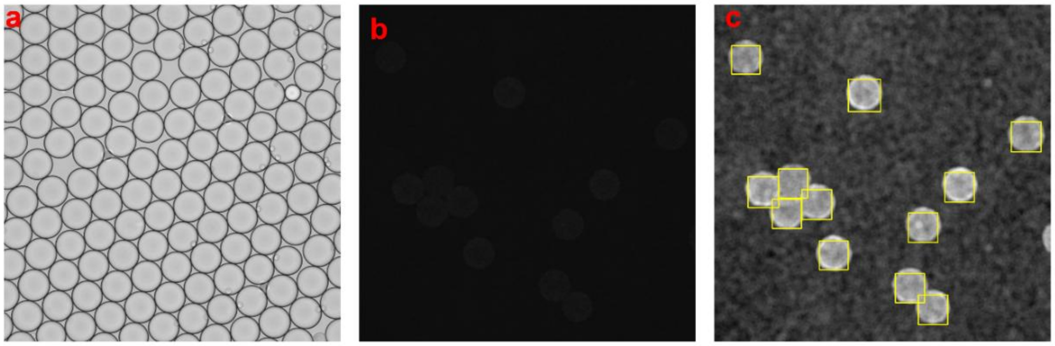

Supplementary figure SF8: depicts the accuracy of GLCM based method by using fluorescently labelled bacteria. (**a**) bright field image, (**b**) fluorescent image of the same field, (**c**) superimposed image of droplets detected by GLCM-image method and fluorescently illuminated image in the same field of view. GLCM-image analysis method gave 0.6% false positives and 0.7% false negatives when compared with the fluorescence-based detection method. *Note:* scales are not relevant in the pictures.
